# Zonisamide improves axial symptoms in dementia with Lewy bodies with parkinsonism: Post hoc analysis of clinical trials^☆^

**DOI:** 10.1016/j.ensci.2021.100384

**Published:** 2021-12-07

**Authors:** Yoshio Tsuboi, Kenji Kochi, Hidenori Maruyama, Yuji Matsumoto

**Affiliations:** a Department of Neurology, Faculty of Medicine, Fukuoka University, Fukuoka, Japan; b Data Science, Sumitomo Dainippon Pharma Co., Ltd., Tokyo, Japan; c Medical Affairs, Sumitomo Dainippon Pharma Co., Ltd., Tokyo, Japan

**Keywords:** Zonisamide, Dementia with Lewy bodies, Axial symptom, DLB, dementia with Lewy bodies, MMSE, mini-mental state examination, PD, Parkinson's disease, PIGD, postural instability and gait disorder, UPDRS, Unified Parkinson's Disease Rating Scale

## Abstract

Patients with dementia with Lewy bodies (DLB) experience worsening axial symptoms with disease progression, which can negatively affect quality of life. Previous phase 2 and 3 clinical trials conducted in Japan showed that zonisamide improved parkinsonism in patients with DLB. In the present study, we performed a post hoc analysis of pooled data from the previous phase 2 and 3 trials to examine the effect of zonisamide on axial symptoms in this patient group. In our pooled analysis, the primary outcome was the change from baseline to 12 weeks in axial symptom score, measured as the sum of Unified Parkinson's Disease Rating Scale Part III items relevant to gait/balance/midline function. A total of 498 patients were included in this analysis. Zonisamide 25 mg and 50 mg significantly reduced the axial symptom score at week 12 compared with placebo (*p* < 0.01 and *p* < 0.001, respectively, by mixed model of repeated measures). Our findings indicate that zonisamide may improve axial symptoms in DLB with parkinsonism and, thus, may potentially reduce the risk of falls and improve quality of life in this vulnerable patient population.

## Highlights

• Worsening axial impairment is a feature of progressive dementia with Lewy bodies.
• Zonisamide may improve axial symptoms in dementia with Lewy bodies.
• Non-dopamine effects of zonisamide may contribute to improvement of axial symptoms.

## 1 Introduction

Dementia with Lewy bodies (DLB) is the second most common neurodegenerative cause of dementia after Alzheimer's disease, accounting for up to 15% of dementia cases [1]. Clinical features of DLB other than dementia include fluctuating cognition, rapid eye movement sleep behavior disorder, recurrent visual hallucinations, and parkinsonism [2], with worsening axial impairment with disease progression [3,4]. Several studies have reported disease-specific gait and/or the presence of axial impairment as important risk factors for falls in patients with Parkinson's disease (PD) and other neurodegenerative diseases, which can lead to a marked decline in the activities of daily living in affected patients and are associated with increased morbidity and mortality in older adults [[5], [6], [7], [8]]. Because DLB and PD have a common pathophysiological background, including nigrostriatal dopaminergic loss, axial symptoms may also be considered a risk for falls in patients with DLB. Improvement in axial symptoms thus represents an unmet need in patients with DLB.

Zonisamide, originally an antiepileptic drug, was previously shown in phase 2 [9] and 3 [10] clinical trials in patients with DLB to improve parkinsonism, assessed by Unified Parkinson's Disease Rating Scale (UPDRS) Part III total score, with a clear benefit for motor function compared with placebo. Based on these outcomes, zonisamide was approved for the treatment of DLB patients with parkinsonism as well as Parkinson's disease in Japan [11]. The proposed pharmacologic mechanisms responsible for the anti-parkinsonian activity of zonisamide against parkinsonism include dopaminergic, non-dopaminergic, and neuroprotective effects [11]. Zonisamide was shown to improve tremor, bradykinesia, and rigidity in DLB with parkinsonism in a recent post hoc analysis of the phase 2 and 3 trial datasets [12]. Given that axial symptoms can be attributed to dysfunction in non-dopaminergic systems [13], we hypothesized that zonisamide may also improve axial symptoms in this patient population. Although postural instability and gait disorder (PIGD; UPDRS Part III items 29 [gait], and 30 [postural stability]) did not significantly decrease in the zonisamide groups in the previous study [12], there are several characteristics and important items pertaining to axial symptoms, in addition to those related to PIGD, which may also be associated with fall risk. Therefore, the current post hoc analysis of pooled data from the phase 2 and phase 3 trials was conducted to examine the effect of zonisamide 25 or 50 mg on axial symptoms as assessed using relevant items from UPDRS Part III.

## 2 Methods

### 2.1 Trial design

**Fig. 1:**
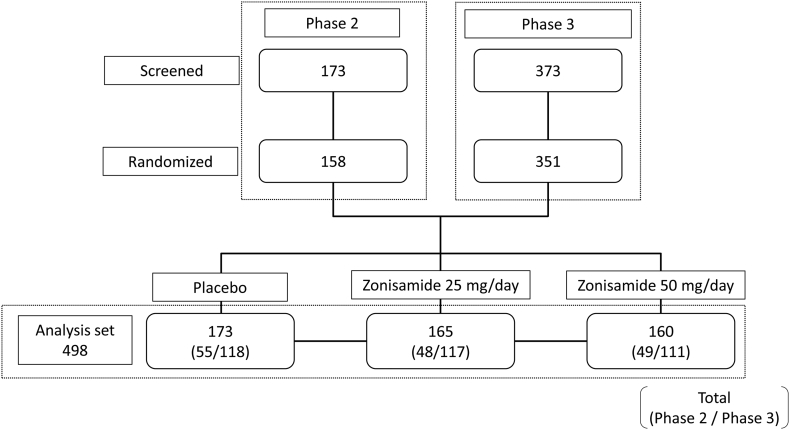
Patient disposition in the phase 2 and phase 3 clinical trials included in the current post hoc analysis. Values in parentheses denote number of patients from the phase 2/phase 3 trials, respectively.

Data were pooled from the phase 2 (JapicCTI-122040) and phase 3 (JapicCTI-152839) trials of zonisamide in patients with DLB conducted in Japan. The design and primary results of these clinical trials have been published previously [9,10]. In brief, both trials featured a 4-week run-in period followed by a 12-week double-blind treatment period. The phase 2 trial included 158 adult patients with DLB who were assigned to receive placebo or zonisamide 25 or 50 mg once daily for 12 weeks. The primary endpoint was the change from baseline in UPDRS Part III total score at week 12. The phase 3 trial enrolled 346 patients and had a similar design, except for the addition of a 40-week open-label extension period at the end of the 12-week double-blind treatment period. The patient disposition for the two clinical trials is shown in Fig. 1. Written informed consent was obtained from all patients prior to enrollment in the included clinical trials, which were conducted in accordance with the principles of the Declaration of Helsinki and were approved by the institutional review board at each participating site.

### 2.2 Patient eligibility

In both clinical trials, eligible patients were adults with probable DLB based on the 2005 version of the clinical diagnostic criteria for DLB [14]. Major inclusion criteria were UPDRS Part III score ≥ 10 and administration of a levodopa/decarboxylase inhibitor for at least 12 weeks prior to screening. In the phase 2 trial, patients with a mini-mental state examination (MMSE) score of 10–26 were eligible for inclusion, whereas those with MMSE score < 10 were excluded from the phase 3 trial. Patients with Parkinson's disease with dementia or Parkinson syndromes other than DLB, patients who were non-responsive to levodopa therapy, patients who had previously received zonisamide, and patients with epilepsy were excluded from the present analysis. The full details of the inclusion and exclusion criteria have been published previously [9,10].

### 2.3 Post hoc analysis

The effect of zonisamide (25 mg and 50 mg) on change from baseline in axial symptom score at 12 weeks was evaluated in the pooled data from the two clinical trials. The axial symptom score was the sum of UPDRS Part III items 18 (speech), 19 (facial expression), 27 (arising from chair), 28 (posture), 29, and 30, representing gait/balance/midline function [15]. The time course of change from baseline in axial symptom score was also evaluated at weeks 4, 8, and 12.

### 2.4 Statistical analysis

All statistical analyses were performed using one-stage meta-analysis methods with individual patient data. Differences in patient demographics and baseline clinical characteristics among the placebo and zonisamide 25 mg and 50 mg groups were evaluated using the Cochran–Mantel–Haenszel test stratified by trial for categorical variables and analysis of variance with trial as the fixed effect for continuous variables. The effect of zonisamide on change from baseline in axial symptom score at 4, 8, and 12 weeks was evaluated using a mixed-effect model for repeated measures among the zonisamide 25 mg, 50 mg, and placebo groups with treatment group, visit, trial, and treatment-by-visit interaction as fixed effects, and baseline value as a covariate. Two-sided *p*-values <0.05 were considered statistically significant in between-group comparisons versus placebo. All statistical analyses were performed using SAS version 9.4 (SAS Institute, Cary, NC, USA).

## 3 Results

### 3.1 Patients

A total of 498 patients were randomized across the two clinical trials. The mean age was 76.6 years, and 277 (55.6%) of the patients were male. Mean DLB duration was 1.4 years, and mean levodopa dose was 262 mg/day. Patient characteristics for the pooled datasets are presented in Table 1. No differences in baseline characteristics were observed among the treatment groups.

**Table 1:** Patient demographics and clinical characteristics at baseline.

	Total (*n* = 498)	Placebo (*n* = 173)	Zonisamide 25 mg (*n* = 165)	Zonisamide 50 mg (*n* = 160)	*p* value*
Male sex	277 (55.6)	99 (57.2)	97 (58.8)	81 (50.6)	0.290
Age, years					
Mean ± SD	76.6 ± 6.7	76.2 ± 7.1	76.4 ± 6.6	77.1 ± 6.4	0.422
≥ 65 years	472 (94.8)	161 (93.1)	157 (95.2)	154 (96.3)	0.427
≥ 75 years	320 (64.3)	108 (62.4)	105 (63.6)	107 (66.9)	0.688
Disease duration					
DLB	1.4 ± 1.7 (*n* = 497)	1.5 ± 1.6	1.4 ± 1.8	1.4 ± 1.6 (*n* = 159)	0.943
Motor dysfunction	3.0 ± 2.5 (*n* = 495)	3.0 ± 2.7 (*n* = 171)	3.0 ± 2.6 (*n* = 164)	3.0 ± 2.3	0.993
Dementia	3.7 ± 2.6 (*n* = 493)	3.7 ± 2.6 (*n* = 172)	3.7 ± 2.6 (*n* = 163)	3.6 ± 2.6 (*n* = 158)	0.935
DLB core feature					
Fluctuating cognition	334 (67.1)	114 (65.9)	110 (66.7)	110 (68.8)	0.851
Visual hallucination	297 (59.6)	101 (58.4)	97 (58.8)	99 (61.9)	0.776
Parkinsonism	498 (100.0)	173 (100.0)	165 (100.0)	160 (100.0)	–
DLB suggestive feature					
REM sleep behavior disorder	235 (47.2)	87 (50.3)	80 (48.5)	68 (42.5)	0.336
Severe neuroleptic sensitivity	55 (11.0)	17 (9.8)	20 (12.1)	18 (11.3)	0.748
Concomitant medication					
Baseline levodopa, mg/day	262 ± 153	257 ± 154	256 ± 159	273 ± 147	0.510
Baseline LEDD, mg/day	295 ± 195	294 ± 209	291 ± 203	300 ± 171	0.923
MAO-B inhibitor	20 (4.0)	7 (4.0)	7 (4.2)	6 (3.8)	0.966
Amantadine	25 (5.0)	6 (3.5)	10 (6.1)	9 (5.6)	0.497
Dopamine agonist	77 (15.5)	27 (15.6)	25 (15.2)	25 (15.6)	0.995
A2A receptor agonist	8 (1.6)	5 (2.9)	2 (1.2)	1 (0.6)	0.213
Droxidopa	21 (4.2)	4 (2.3)	6 (3.6)	11 (6.9)	0.107
Anticholinergic drug	7 (1.4)	2 (1.2)	1 (0.6)	4 (2.5)	0.330
COMT inhibitor	24 (4.8)	13 (7.5)	5 (3.0)	6 (3.8)	0.120
Antidementia drug	361 (72.5)	122 (70.5)	125 (75.8)	114 (71.3)	0.500
Yokukansan	95 (19.1)	35 (20.2)	31 (18.8)	29 (18.1)	0.891
Other CNS drugs	207 (41.6)	68 (39.3)	74 (44.8)	65 (40.6)	0.579
Baseline total score					
UPDRS Part III	31.5 ± 11.5	30.8 ± 10.6	32.3 ± 12.5	31.6 ± 11.8	0.471

Data are presented as mean ± SD or n (%). *Analysis of variance for continuous variables and Cochran–Mantel–Haenszel test for categorical variables. SD, standard deviation; DLB, dementia with Lewy bodies; REM, rapid eye movement; LEDD, levodopa equivalent daily dose; MAO-B, monoamine oxidase-B; A2A, adenosine A2A; COMT, catechol-*O*-methyltransferase; CNS, central nervous system; UPDRS, Unified Parkinson's Disease Rating Scale.

### 3.2 Outcomes

**Fig. 2:**
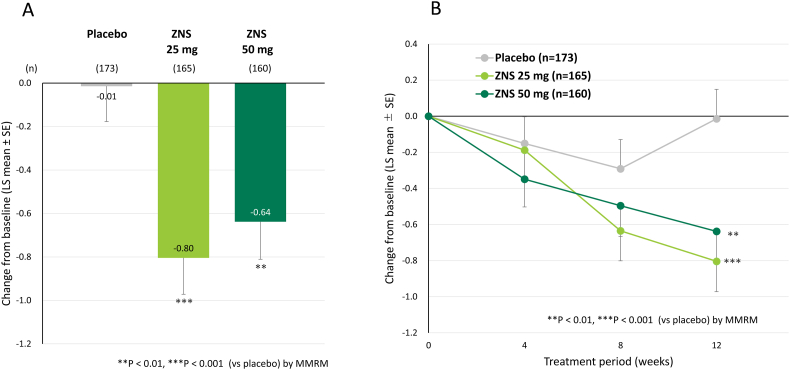
Change from baseline in axial symptom score at week 12. LS mean, least-square mean; MMRM, mixed model of repeated measures; SE, standard error; ZNS, zonisamide.

Both doses of zonisamide reduced the axial symptom score at week 12 compared with placebo (Fig. 2). The mean (± standard deviation) baseline axial symptom scores in the placebo, zonisamide 25 mg, and zonisamide 50 mg groups were 7.79 (± 3.38), 8.41 (± 3.91), and 8.38 (± 3.61), respectively. At week 12, the change in axial symptom score from baseline was −0.01 (± 0.16; least squares mean ± standard error), −0.80 (± 0.17; *p* = 0.0006), and − 0.64 (± 0.17; *p* = 0.0076) for placebo, zonisamide 25 mg, and zonisamide 50 mg, respectively. In the time course analysis, the axial symptom scores in the zonisamide groups were decreased at week 4 and continued to decrease until week 12. In the placebo group, the axial symptom score was decreased at weeks 4 and 8, but the score at week 12 did not differ from the baseline score.

## 4 Discussion

The results of this post hoc analysis indicate that zonisamide 25 mg and 50 mg decreased the axial symptom score compared with placebo and may therefore improve axial symptoms in DLB with parkinsonism. Although PIGD alone did not significantly decrease with zonisamide in a previous report [12], in this study, zonisamide improved overall axial symptoms as assessed by PIGD- and other axial symptom-related items in UPDRS Part III. Improving axial symptoms may contribute to the reduction of frequency of falls, and no clinical trial to date has evaluated the effect of zonisamide on axial symptoms in patients with DLB.

Axial symptoms have previously been reported to be predominantly mediated by non-dopaminergic systems in Parkinson's disease [13]; therefore, although it is not clear whether axial symptoms increase the risk of falls in patients with DLB, a similar mechanism might be expected in those patients. The proposed pharmacologic mechanisms responsible for the anti-parkinsonian activity of zonisamide include both dopaminergic (activation of dopamine synthesis and release and inhibition of monoamine oxidase-B) and non-dopaminergic (blockade of sodium channels and T-type calcium channels) functions [11]. In a recent randomized cross-over trial, an improvement in axial symptoms was observed in PD patients who underwent optimized deep brain stimulation of the subthalamic nucleus [16], a relay nucleus of the indirect striato-pallidal pathway [17]. Findings from a previous pre-clinical study indicate that zonisamide does not affect the direct pathway but instead inhibits the indirect striato-pallidal pathway, which is mediated by the δ_1_ receptor, to exert its anti-parkinsonian action [18]. The results of the present analysis may therefore be attributable to non-dopaminergic mechanisms of zonisamide, including stimulation of the indirect striato-pallidal pathway, resulting in improvements in axial symptoms.

### 4.1 Limitations

The current analysis was not prespecified and was conducted post hoc. The analysis is further limited by the relatively small sample sizes of the trial datasets, inclusion of only Japanese patients, and short trial durations, meaning that further prospective evidence from larger global studies is needed to confirm the generalizability of these results.

### 4.2 Conclusions

The results of the present post hoc analysis indicate that zonisamide as adjunct therapy to levodopa may improve axial symptoms in DLB with parkinsonism; further studies are needed to confirm whether zonisamide may represent a potential therapeutic option to reduce the risk of falls in this patient population.

## Funding

This work was funded by Sumitomo Dainippon Pharma Co., Ltd.

## Data sharing statement

The datasets used in the current analysis are not available for sharing at present, but will be available upon reasonable request via the Clinical Study Data Request site (https://www.clinicalstudydatarequest.com/Study-Sponsors.aspx). Access may be provided after a research proposal is submitted and has received approval from the Independent Review Panel and after a Data Sharing Agreement is in place.

## Contributor statements

YT, HM, and YM contributed to the study conception and design, and wrote the manuscript. KK was involved in the design of the study and data analysis. All authors interpreted the data, and discussed and agreed on the content of the manuscript prior to submission.
